# Genome analysis reveals three genomospecies in *Mycobacterium abscessus*

**DOI:** 10.1186/1471-2164-15-359

**Published:** 2014-05-12

**Authors:** Mohamed Sassi, Michel Drancourt

**Affiliations:** Aix Marseille Université, URMITE, UMR63, CNRS 7278, IRD 198, Inserm, 1095 Marseille, France; Unité de Recherche sur les Maladies Infectieuses et Tropicales Emergentes, Faculté de Médecine, 27, Boulevard Jean Moulin, Marseille cedex 5, France

**Keywords:** *Mycobacterium abscessus*, *Mycobacterium bolletii*, *Mycobacterium massiliense*, Araucaria, Mycobacteriophage, Prophage

## Abstract

**Background:**

*Mycobacterium abscessus* complex, the third most frequent mycobacterial complex responsible for community- and health care-associated infections in developed countries, comprises of *M. abscessus* subsp. *abscessus* and *M. abscessus* subsp. *bolletii* reviously referred as *Mycobacterium bolletii* and *Mycobacterium massiliense*. The diversity of this group of opportunistic pathogens is poorly described.

**Results:**

In-depth analysis of 14 published *M. abscessus* complex genomes found a pan-genome of 6,153 proteins and core-genome of 3,947 (64.1%) proteins, indicating a non-conservative genome. Analysing the average percentage of amino-acid sequence identity (from 94.19% to 98.58%) discriminates three main clusters C1, C2 and C3: C1 comprises strains belonging to *M. abscessus*, C2 comprises strains belonging to *M. massiliense* and C3 comprises strains belonging to *M. bolletii*; and two sub-clusters in clusters C2 and C3. The phylogenomic network confirms these three clusters. The genome length (from 4.8 to 5.51-Mb) varies from 5.07-Mb in C1, 4.89-Mb in C2A, 5.01-Mb in C2B and 5.28-Mb in C3. The mean number of prophage regions (from 0 to 7) is 2 in C1; 1.33 in C2A; 3.5 in C2B and five in C3. A total of 36 genes are uniquely present in C1, 15 in C2 and 15 in C3. These genes could be used for the detection and identification of organisms in each cluster. Further, the mean number of host-interaction factors (including PE, PPE, LpqH, MCE, Yrbe and type VII secretion system ESX3 and ESX4) varies from 70 in cluster C1, 80 in cluster C2A, 74 in cluster C2B and 93 in clusters C3A and C3B. No significant differences in antibiotic resistance genes were observed between clusters, in contrast to previously reported in-vitro patterns of drug resistance. They encode both penicillin-binding proteins targeted by β-lactam antibiotics and an Ambler class A β-lactamase for which inhibitors exist.

**Conclusions:**

Our comparative analysis indicates that *M. abscessus* complex comprises three genomospecies, corresponding to *M. abscessus, M. bolletii*, and *M. massiliense*. The genomics data here reported indicate differences in virulence of medical interest; and suggest targets for the refined detection and identification of *M. abscessus*.

**Electronic supplementary material:**

The online version of this article (doi:10.1186/1471-2164-15-359) contains supplementary material, which is available to authorized users.

## Background

The non-tuberculous mycobacterium *Mycobacterium abscessus* was long confused with *Mycobacterium chelonae*[1]. Other closely related species include *Mycobacterium salmoniphilum*[2], *Mycobacterium immunogenum*[3], *Mycobacterium massiliense*[4], *Mycobacterium bolletii*[5] and *Mycobacterium franklinii*[6] altogether forming the *Mycobacterium chelonae-abscessus* complex. This complex is the third most frequent mycobacterial complex infecting humans in developed countries besides the *Mycobacterium tuberculosis* and *Mycobacterium avium* complexes [7, 8]. Bibliometrics retrieving over 1,700 publications in the Medline database illustrates the fact that this complex is emerging, causing both sporadic cases and outbreaks of community-acquired and health-care associated infections [9]. Not only humans but also cats [10, 11] and dolphins [12–14] are infected while fishes are uniquely infected by *M. salmoniphilum*[2, 15].

Current nomenclature is that the species *M. abscessus* comprises two subspecies named *M. abscessus* subsp. *abscessus* and *M. abscessus* subsp. *bolletii*. Later taxon accommodates isolates previously referred as *M. bolletii* or *M. massiliense*[16]. This nomenclature however may obscure the true diversity of mycobacteria in this complex. While the 16S rRNA gene yields an identical sequence for *M. abscessus* and *M. bolletii*, it shares 99% sequence identity with *M. massiliense. Rpo*B gene sequencing founded the description of recent species [17–19] but yielded further conflicting results [20–22]. Multilocus sequencing analysis [23] and multispacer sequence typing [24] differentiated *M. massiliense* from *M. bolletii*. In this report, the previous nomenclature *M. abscessus*, *M. bolletii* and *M. massiliense* forming the *M. abscessus* complex, has been retained for clarity.

The availability of 39 *M. abscessus*, 13 *M. massiliense* and two *M. bolletii* genomes in the National Center for BioInformatics (NCBI) genome database provides new opportunities to assess the diversity of this species. Here, we review 14 complete published *M. abscessus* complex genomes and compare them with the re-annotated *M. tuberculosis* H37Rv genome (Table 1) in order to in-depth analyse the diversity of *M. abscessus*.

**Table 1:** **List of** ***Mycobacterium abscessus*** **genomes here studied**

Clusters	Organism	Isolated from	Geography	BioProject
**C1**	***M. abscessus*** **Type strain**	human knee infection	United States	PRJNA61613, PRJNA15691
**C1**	***M. abscessus*** **M93**	sputum sample from a Malaysian patient presenting with a prolonged productive cough suggestive of a bacterial lower respiratory tract infection	Malysia	PRJNA180393, PRJNA84203
**C1**	***M. abscessus*** **M94**	sputum sample of a Malaysian patient with a persistent cough and fever and consolidation in the chest radiograph	Malysia	PRJNA180394, PRJNA88149
**C1**	***M. abscessus*** **M152**	acid-fast bacillus positive sputum of a Malaysian man	Malysia	PRJNA159789
**C1**	***M. massiliense*** **strain GO 06**	undergone knee joint surgery	Brazil	PRJNA170732, PRJNA168263
**C2A**	***M. massiliense*** **Type strain**	sputum specimen from hemoptoic pneumonia	Marseille	PRJNA180742, PRJNA65215
**C2A**	***M. massiliense*** **M18**	lymph node biopsy specimen from a Malaysian patient suspected of having tuberculous cervical lymphadenitis	Malysia	PRJNA89593
**C2A**	***M. massiliense*** **M154**	bronchoalveolar lavage fluid of a Malaysian patient presenting with lower respiratory tract infection	Malysia	PRJNA89603
**C2B**	***M. abscessus*** **47 J26**	sputum sample from a patient with Cystis fibrosis	England	PRJNA179981, PRJNA73255
**C2B**	***M. abscessus*** **M115**	sputum from a Malaysian patient presenting with persistent cough and loss of body weight suggestive of pulmonary tuberculosis	Malysia	PRJNA89601
**C2B**	***M. abscessus*** **M139**	sputum sample of a 26-year-old Nepalese male presenting with hemoptysis	Nepal	PRJNA159701
**C2B**	***M. abscessus*** **M172**	putum isolate from a Malaysian patient	Malysia	PRJNA89599
**C3A**	***M. bolletii*** **Type strain**	respiratory tract specimen collected in woman with hemoptoic pneumonia	Marseille	PRJNA180015, PRJNA73695
**C3B**	***M. abscessus*** **M24**	the bronchoalveolar lavage fluid of a Malaysian patient	Malysia	PRJNA89595

## Results and discussion

### *M. abscessus* complex pan- and core-genome

*M. abscessus* complex genomes comprise one circular chromosome. In addition, *M. abscessus* ATCC 19977 contains one 23-kb plasmid identical to the *Mycobacterium marinum* pMM23 plasmid, encoding mer operon and mercury reductase protein, which may confer resistance to organo-mercury compounds [25]. In order to normalize the predicted proteins and to minimize the differences of presence/absence of genes and length, coding sequences were predicted using prodigal software [26]. We identified a total of 70,309 protein-coding sequences which number varies from 4,651 to 5,079 in each genome (Table 2). The core-genome contains 57,172 protein sequences accounting for 64.15% of the pan-genome. This figure indicates a non-conservative genome contrary to that of *Mycobacterium tuberculosis*, a conservative-genome pathogen which core-genome accounts for 96.1% of the pan-genome [27]. Using orthoMCL [28] with a conservative parameter value of 50% sequence identity, we categorized these 70,309 proteins into 6,153 orthologous protein groups, including 3,947 core-genome groups and 55 strain-specific groups.

**Table 2:** ***Mycobacterium abscessus*** **core genome and unique genes**

Clusters	Organism	CDS	Unique core genome	Total genes
	***M. abscessus***	**-**	**36**	**180**
**C1**	*M. abscessus* T	4954	-	-
	strain GO 06	4944	-	-
	M93	4733	11	11
	M94	4841	10	10
	M152	4762	-	-
	***M. massiliense***	**-**	**15**	**107**
**C2A**	*M. massiliense* T	4962	3	3
	M18	4663	8	8
	M154	4651	-	-
**C2B**	47 J26	4766	-	-
	M115	4802	4	4
	M139	4754	4	4
	M172	5079	20	20
	***M. bolletii***	**-**	**15**	**30**
**C3A**	*M. bolletii* T	4733	9	9
**C3B**	M24	4960	23	23
	***M. abscessus*** **core genome**	**-**	**3,947**	**57,172**

### *M. abscessus* complex diversity

The average percentage of amino-acid sequence identity (AAI) of core proteins was determined as previously described [29]. The AAI values indicate that *M. abscessus* complex forms three main clusters: cluster 1 (C1) includes *M. abscessus* type strain and strains M93, 94, M152 and Go06; cluster 2 (C2) contains two subclusters: cluster 2A (C2A) includes *M. massiliense* type strain and strains M154 and M18; cluster 2B (C2B) includes strains 47 J26, M115, M172 and M139; cluster 3 (C3) includes two subclusters: cluster 3A (C3A) includes *M. bolletii* type strain and cluster 3B (C3B) includes *M. bolletii* strain M24 (Table 3).

**Table 3:** **Average nucleodite identity and characteristics of** ***Mycobacterium abscessus*** **genomes**

Clusters		Strains	Genome lenght Mb	Genome GC%	AAI Vs ***M. abscessus*** T	AAI Vs ***M. bolletii*** T	AAI Vs ***massiliense*** T
***M. abscessus***	**C1**	***M. abscessus T***	5,09	62,7	100,00	95,56	94,74
		**M93**	5,08	64,2	97,30	95,35	94,76
		**M94**	5,1	64,2	97,56	95,67	94,79
		**M152**	4,91	64,1	98,59	96,33	95,73
		**strain GO 06**	5,07	64,2	98,35	95,23	95,64
***M. massiliense***	**C2A**	***M. massiliense T***	5,2	64,2	95,56	96,13	100,00
		**M18**	4,89	64,2	96,66	96,09	97,57
		**M154**	4,8	64,1	96,14	95,81	97,26
	**C2B**	**M115**	4,98	64,1	96,16	95,36	96,92
		**M172**	5,2	64,2	95,30	94,93	96,17
		**M47 J26**	4,87	64,1	96,23	95,74	96,93
		**M139**	5,05	64,1	95,94	95,64	96,88
***M. bolletii***	**C3A**	***M. bolletii T***	5,05	64,2	94,51	100,00	95,33
	**C3B**	**M24**	5,51	64,2	94,91	96,47	94,20

**Figure 1:**
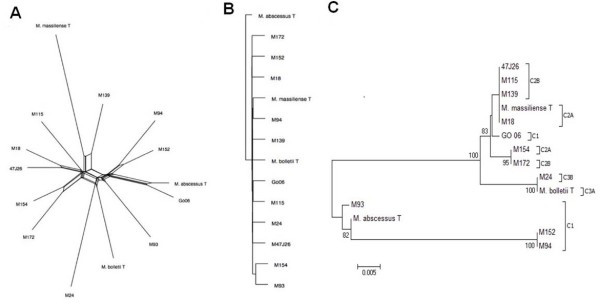
**Phylogenomic analysis of** ***M. abscessus*** **. A**. Aligned whole genomes phylogenetic network. **B**. Gene content phylogeny constructed from the matrix of discrete characters using the neighbor-joining method. **C**. *Rpo*B gene based phylogentic tree using neighbor-joining method.

*M. abscessus* complex proteomes were further aligned using Mauve software [30] to infer phylogeny using the Neighbor-Net algorithm in the package SplitsTree4 [31]. The phylogenomic network confirms the three clusters C1, C2 and C3 (Figure 1A). A phylogenomic tree based on gene content (i.e., the presence or absence of orthologs) (Figure 1B) organizes *M. abscessus* differently from the whole genome concatenated tree (Figure 1A) or even the phylogenetic tree based on *rpo*B gene sequence (Figure 1C). Phylogenomic analysis indicates that the *M. abscessus* gene repertoires have different evolutionary histories and suggests that differential gene loss and lateral gene acquisition are playing important roles in the evolution of some *M. abscessus* strains. Notably, the situation of strain Go06 is confusing, as it presents 98.4% AAI with *M. abscessus* type strain in C1 (Figure 1A) whereas its *rpo*B gene sequence and single nucleotide polymorphisms analysis are indicative of *M. massiliense*[8, 32]. Our analyses indicate that strain Go06 have an ambiguous classification as a chimera between *M. abscessus* and *M. massiliense* and is the only example compatible with a lateral transfer of *rpo*B gene.

**Figure 2:**
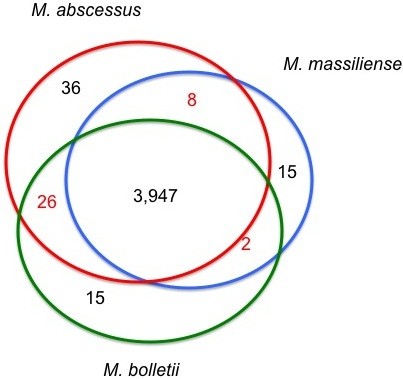
**Core genomes in** ***M. abscessus*** **clusters.**

Functional analysis using Clusters of Orthologous Groups database (COG) [33] and BLASTP indicates unique genes in C1, encoding hypothetical proteins, proteins implicated in transcription, energy production and transport, carbohydrate metabolism and transport, lipid metabolism, nucleotide metabolism and transport, amino-acid metabolism and transport, post-translational modification and inorganic ion transport and metabolism (Table 2, Figure 2). Within C2, unique genes encode hypothetical proteins, proteins implicated in transcription and lipid metabolism. Within C3, unique genes encode hypothetical proteins, proteins implicated in amino-acid metabolism and transport and translation. These unique genes could base a refined identification of the three genomospecies. However, we could not exclude that these unique genes could be due to a coding sequence, which arose de novo, to HGT or gene loss for the other subspecies. In the case of absence of a gene, this could also be due to a real loss or to an assembly artefact.

C1 strains have been isolated from American and Malaysian patients suffering knee infection and lower respiratory infection, respectively (Table 1). C2A strains were isolated from Malaysian and French patients suffering severe, respiratory tract infections. C2B strains were isolated from Nepalese, Malaysian and English patients suffering respiratory tract infections, including cystis fibrosis and pulmonary tuberculosis patients. C3A and C3B strains were exclusively isolated from patients suffering respiratory tract infections, in France and Malaysia, respectively. Therefore, clusters specify the clinical form and geographical origin of the infection.

Altogether, genomics analyses revealed a more heterogeneous structure of *M. abscessus* complex than the one currently suggested by the nomenclature, which recognizes only two subspecies within *M.* absc*essus*[16]. It has been proposed that two genomes exhibiting AAI >96% belong to the same species [34, 35]. Therefore, AAI analysis indicates that *M. abscessus* is in fact comprising of three genomospecies, corresponding to previous nomenclature of *M. abscessus* (C1), *M. massiliense* (C2) and *M. bolletii* (C3). Using an AAI <97% threshold would further determine two subspecies in *M. massiliense* (C2A and C2B) and in *M. bolletii* (C3A and C3B). Recent whole genome sequencing analyses of clinical isolates in Great Britain also clearly distinguished three clusters in agreement with the three here reported [8]. All these data support revaluating the taxonomy of *M. abscessus* complex, to recognize three genomospecies *M. abscessus* (C1), *M. bolletii* (C2), and *M. massiliense* (C3); and four unnamed subspecies C2A, C2B; C3A, C3B.

### *M. abscessus* prophagome

*M. abscessus* median GC% content is 64.2%, ranging from 62.7% (*M. abscessus* ATCC 19977) to 64.2% (strain Go 06). The GC% is not characteristic of the clusters as the median GC% content of C1, C2A and C3 is 64.2%, close to the median 64.1% GC% content in C2B.

**Figure 3:**
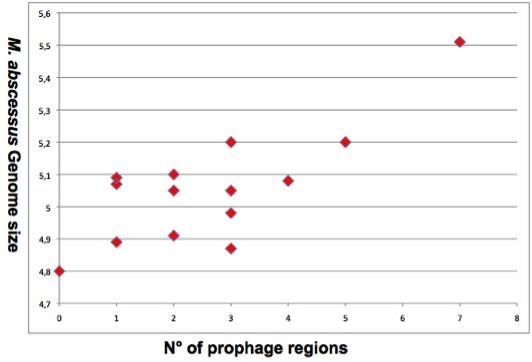
**Correlation between** ***Mycobacterium abscessus*** **genomes size (y axis) and the number of prophages (x axis).**

However, there is a significant 14.7% variation in the genome length from 4.8-Mb (*M. abscessus* M154) to 5.51-Mb (*M. abscessus* M24) with a median of 5.07-Mb. The median of genome size is 5.07-Mb in C1, 4.89-Mb in C2A, 5.01-Mb in C2B and 5.28-Mb in C3. Differences in the genome size correlate with the number of prophage regions which are detected in 13/14 *M. abscessus* genomes (Figure 3): *M. abscessus* M154 (*M. massiliense* C2A) has the smallest genome encoding no prophage whereas *M. bolletii* M24 (C3) has the largest genome encoding seven prophage regions (Figure 3). Prophage regions comprise up to 5% of the genome lenght in *M. abscessus* M172. The number of prophage regions in other genomes is diverse, ranging from one to six regions encoding putative genes in the subsystem of phages, prophages, transposable elements and plasmids, which might contribute to species diversity [36]. The mean number of prophage regions is 2 in C1, 1.33 in C2A, 3.5 in C2B and 5 in C3. This observation confirms the particularity of C3: *M. bolletii* CIP108541^T^ contains a 13-kb and a 63-kb prophage whereas *M. bolletii* strain M24 contains seven prophage regions including one 17-kb region homologous to the *M. bolletii* CIP108541^T^ 13-kb region and a 27-kb region homologous to the *M. massiliense* CCUG 48898 50-kb region [37, 38] (Table 4). *M. abscessus* genomes encode putative phage-related genes necessary for phage replication as well as phage-tail protein, phage endolysin, capsid proteins (major protein and scaffold proteins) and phage tape measure protein. Both ends of this region encode putative phage integrases. *M. abscessus* genomes encode small prophage-like regions. However, only *M. bolletii* has been reported to produce a mycobacteriophage that we named Araucaria after we recently resolved its electron microscopy 3D structure [39]. *M. abscessus* M94 genome harbours one particular pseudo-tRNA spanning the region 51,150-57,394 in contig 33, which is not observed in the other *M. abscessus* genomes [40]. Phages have been reported to increase virulence of their host and encode antimicrobial resistance genes [41]. In *M. abscessus* however, no such genes were identified but phages could be targeted for the differentiation between the three *M. abscessus* genomospecies.

**Table 4:** ***Mycobacterium abscessus*** **prophages**

Clusters		Strain	Region	Region_length	CDS	Region_position
***M. abscessus***	**C1**	***M. abscessus T***	1	81 Kb	110	1754551-1835095
		***M93***	1	16.4 Kb	33	197463-213867
			2	38 Kb	51	232006-270072
			3	53 Kb	70	1762720-1815780
			4	20.2 Kb	26	1820768-1841058
		**M94**	1	58.3 Kb	84	1039523-1097850
			2	79.4 Kb	99	4959719-5039151
		**M152**	1	48.9 Kb	53	1897722-1946683
			2	34.9 Kb	44	4784847-4819818
		**Go06**	1	65 Kb	44	1768158-1833157
***M. massiliense***	**C2A**	***M. massiliense T***	1	12.5 Kb	21	1600973-1613514
			2	31.3 Kb	33	1620002-1651385
			3	50.4 Kb	69	3907205-3957680
		**M18**	1	62.8 Kb	67	4702725-4765592
		**M154**	0	0	0	0
	**C2B**	**M115**	1	11.6 Kb	10	1416841-1428481
			2	77.1 Kb	102	1624644-1701770
			3	55.3 Kb	79	3356346-3411651
		**M172**	1	55.1 Kb	74	502478-557677
			2	50.7 Kb	50	546109-596832
			3	59 Kb	67	1934186-1993225
			4	31.1 Kb	33	2050376-2081567
			5	39.4 Kb	45	3711805-3751246
			6	19.6 Kb	40	3753466-3773078
		**M47J26**	1	39.9 Kb	48	1066714-1106668
			2	12.4 Kb	16	3596408-3608873
			3	41.4 Kb	42	3823414-3864899
		**M139**	1	35.9 Kb	43	2906235-2942215
			2	12.5 Kb	17	5033731-5046263
***M. bolletii***	**C3A**	***M. bolletii T***	1	41.6 Kb	47	1684736-1726377
			2	20.9 Kb	38	1727918-1748849
			3	12.4 Kb	16	3641720-3654182
	**C3B**	**M24**	1	37.1 Kb	51	560940-598047
			2	37 Kb	37	1680197-1717263
			3	17 Kb	21	3830340-3847343
			4	18.1 Kb	34	5051771-5069955
			5	26 Kb	35	5155113-5181190
			6	19.2 Kb	26	5213195-5232444
			7	26.5 Kb	33	5312024-5338593

### *M. abscessus* complex resistome

As all mycobacteria, *M. abscessus* complex is embedded into a hydrophobic cell wall barrier to hydrophilic antibiotics. Accordingly, *M. abscessus* is multidrug resistant organisms exhibiting different drug resistance [42–44]. *M. abscessus* genomes encode many proteins potentially involved in drug-efflux systems, including members of the major facilitator family, ABC transporters and MmpL proteins; Small Multidrug Resistance-family, a family of lipophilic drug efflux proteins [45]; and a multidrug resistance stp protein similar to *M. tuberculosis* involved in spectinomycin and tetracycline resistance [46]. *M. abscessus*, *M. bolletii* and *M. massiliense* were reported to be *in-vitro* susceptible to amikacin; however, comparison with the *M. tuberculosis* H37Rv resistome and the antibiotic resistance databases indicate that *M. abscessus* encodes an aminoglycoside 29-N-acetyltransferase and aminoglycoside phosphotransferases involved in resistance to aminoglycosides. Also, genetic analyses disclosed 16S rRNA gene mutations conferring aminoglycoside resistance [4, 5, 47]. Indeed, the presence of a single rRNA operon in all of the *M. abscessus* genomes favours the occurrence of dominant mutations conferring resistance to aminoglycosides and macrolides. *M. abscessus* genomes encode a rifampin ADP-ribosyl transferase and monooxygenases potentially involved in resistance to rifampin and tetracyclines. Moreover, *M. abscessus* genomes encode three *tet*(M) genes conferring resistance to tetracyclyine and doxycycline; the number of *tet*(M) genes was correlated to the resistance to cyclines in *Escherichia coli*[48]. However, *M. massiliense* was reported to be susceptible and *M. abscessus* and *M. bolletii* to be resistant to doxycycline [49]. *M. abscessus* genomes encode resistance to fusidic acid, glycopeptides, MLS (Macrolide-Lincosamide-StreptograminB), phenicols, rifampicin, sulphonamide and trimethoprim. Also, *M. abscessus* genomes encode FolP homologs conferring resistance to cotrimoxazole, homolog of UDP-N- acetylglucosamine 1-carboxyvinyltransferase, a MurA protein conferring resistance to fosfomycin and homologs of 23S rRNA methylases conferring resistance to macrolides. Also, *M. abscessus* genome encodes an erm(41) gene which mutations were reported to confer clarithromycin resistance [50]. *In-vitro* tests showed that *M. massiliense* clinical isolates could be distinguished from *M. abscessus* isolates for their susceptibility to ciprofloxacin [51] whereas *M. bolletii* isolates were reported to be resistant to all quinolones [52]. A mutation at codon 90 in gyrA gene was reported in clinical isolates of *M. abscessus* exhibiting high resistance to ciprofloxacin [51]. This observation contrasts with our genome analysis, which found no such mutations, suggesting that other mechanisms of resistance may be involved in high-level resistance to quinolones [52]. Accordingly, we found that *M. abscessus* mycobacteria encode qepA2, a plasmidic gene conferring quinolone resistance in gram-negative bacteria [53]. *M. abscessus* mycobacteria were reported to be *in-vitro* resistant to penicillin, amoxicillin, cefoxitin, ceftriaxone, cefotaxime and imipenen [4, 5]. This contrasts with the fact that they encode Penicillin-binding proteins (PBPs), targets for β-lactam antibiotics (except for tabtoxinine-β-lactam, which inhibits glutamine synthetase), which are essential for peptidoglycan synthesis [54, 55]. *M. abscessus* genomes encode an Ambler class A β-lactamase homologous to β-lactamases in gram-negative bacteria and to two β-lactamases in *M. tuberculosis*. β-lactamases inhibitors have not been evaluated against *M. abscessus sensu lato* mycobacteria.

### Genome-based analysis of host-interactions

**Figure 4:**
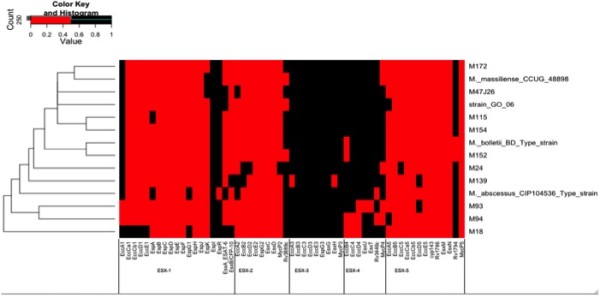
**Heatmap clusterisation of** ***Mycobacterium abscessus*** **type VII secretion system compared to** ***Mycobacterium tuberculosis*** **H37Rv.** *M. abscessus* strains are listed on the left side of the map.

*M. abscessus* are ubiquitous environmental organisms in soil and water [9] where they may have to cope with amoeba: *M. chelonae, M. abscessus, M. massiliense* and *M. immunogenum* were reported to survive within *Acanthamoeba polyphaga* tropohozoites and cysts [5]. Accordingly, our analyses indicate that *M. abscessus* genomes encode factors implicated in host interactions. The mean number of genes encoding proline-glutamate (PE), proline-proline glutamate (PPE), 10-kDa lipoprotein antigen precursor (LpqH), Mammalian Cell Entry (MCE), oxidoreductase (Yrbe) and type VII secretion system is of 70 in C1, 80 in C2A, 74 in C2B and 93 in C3. In *M. abscessus*, rough colonies lack *mmp*L4 (a gene required for glycopeptidolipid biosynthesis) and lost surface colonization, replication into human macrophages and stimulation of innate immune response; these observations suggested that glycopeptidolipid was a virulence factor [56–58]. Accordingly, glycopeptidolipids are required for sliding motility [59] and biofilm formation [60]. Glycopeptidolipids have also been suspected to inhibit phagocytosis of *M. avium* subsp*. avium*[61]. *M. abscessus* genomes encode MCE proteins similar to *M. tuberculosis* H37Rv. MCE operon promotes internalization of *M. tuberculosis* by mammalian cells [62] and initiates rapid induction of transcription of genes involved in substrate trafficking [63]. The number of *mce* operons which correlated with pathogenicity [64], varies from six in C2B to 13 in C3. In parallel, *M. abscessus* genomes encode 12 (C1) to 21 (C3A, C3B) copies of Yrbe proteins. As for secretion systems, recent evidences showed that mycobacteria evolved specialized type VII secretion systems to transport extracellular proteins across the cell wall [65]. Type VII secretion systems ESX-1 and ESX-5 are involved in cell-to-cell migration of *M. tuberculosis*[65, 66]. In *M. abscessus*, our analyses indicate that ESX-3 and ESX-4 systems are conserved (Figure 4). However, *M. abscessus* M139 (C2B) lacks two proteins of the ESX-3 system and *M. abscessus* M93 (C1) lacks ESAT-6 like and CFP-10-like proteins secreted by the ESX-4 system. Interestingly, *M. abscessus* M18 (C2A) encodes ESAT-6 and CFP-10 proteins secreted by ESX-1 system. In addition, there are two or three PE and six (*M. massiliense*, *M. abscessus* M115 or *M. abscessus* 47 J26) to 12 (*M. bolletii* M24) PPE proteins, which are reported to be involved in the virulence of *M. tuberculosis*[67]. Our analyses further indicated that proteins related to phenazine biosynthesis, homogentisate catabolism, phenylacetic acid degradation and DNA degradation might have been transferred from *Actinobacteria* (e.g. *Rhodococcus* spp., *Streptomyces* spp.) and pseudomonas (*Pseudomonas aeruginosa* and *Burkholderia cepacia*). Although distantly related, these bacteria share the same ecosystem as *M. abscessus* within cystic fibrosis microbiota.

## Conclusions

Our in-depth genomic analyses indicate that *M. abscessus* has a non-conservative genome, suggesting the possibility of on-going transfer of additional genetic material. Unsurprisingly, *M. abscessus* has already acquired antibiotic resistance. Also, phages have mediated diversity and horizontal gene transfer which drived the rapid evolution of this complex. Indeed, gene transfers have driven the evolution of *M. abscessus* towards three different genomospecies *M. abscessus*, *M. massiliense* and *M. bolletii*; and the evolution of four different yet unnamed subspecies. Each genomospecies has its own specificities in terms of genome size, prophagome and genome content. We identified 66 genes uniquely present in each genomospecies; these genes could be used in refined detection and identification of *M. abscessus* organisms. These genomic differences support differences in host interactions and the clinical presentation of infection with *M. massiliense* (C2A and C2B) being more virulent than the two other genomospecies. Host-interaction factors may contribute to the ability of *M. abscessus* to colonize mammalian hosts where its respiratory tract habitat put it in close proximity to other serious opportunist pathogens which can act as donors of additional host-interaction factors.

Here reported informations regarding differences between *M. abscessus* genomespecies will help understanding their pathogenesis factors and could reveal new, more specific targets for drug design and diagnosis tools.

## Methods

### Genome dataset

The whole genomes of 14 *M. abscessus* strains were downloaded from Genbank (Table 1). The genomic sequence, either contigs or finished genomes were concatenated to one pseudogenome per genome.

### Prophage detection and genome annotation

Protein sequences were predicted using prodigal software [26] to generate normalized files containing the combined protein sequences of all 14 genomes. Prophage regions were detected using PHAST software (Table 4). Predicted proteins were annotated using BLASTp against the National Center for Biotechnology Information (NCBI) non-redundant (NR) database, UNIPROT (http://www.uniprot.org/), the Clusters of Orthologous Groups (COG) [33] and a home-made antibiotic resistance gene database.

### Genome clustering and calculation of core genomes

Proteome sequences were compared using by BlastP and pairwise alignments using ClustalW and the ANI was determined by the mean percentage of nucleotide sequence identity of core proteins [29]. We clustered the *M. abscessus* homologous genes using orthoMCL [28] on the translated protein sequences of all predicted genes with a conservative parameter value of 50% sequence identity. The determination of the different unique core genomes was based on the homology clusters found by orthoMCL.

### Phylogenetic analysis

*M. abscessus* proteomes were aligned using Mauve software [30] to infer phylogeny using the Neighbor-Net algorithm in the package SplitsTree4 [31]. The orthologous group data found by orthoMCL were used to construct a whole-genome phylogenetic tree based on gene content. We generated a matrix of binary discrete characters (“0” and “1” for absence and presence, respectively) [68]. Using this matrix, we constructed a phylogenetic tree implementing the neighbor-joining (NJ) method within SplitsTree4 [31].

### Availability of supporting data

The data set of Figure 1C supporting the results of this article is available in the TreeBase (http://treebase.org/treebase-web/home.html) repository, under the accession URL http://purl.org/phylo/treebase/phylows/study/TB2:S15632.

Reviewer access URL: http://purl.org/phylo/treebase/phylows/study/TB2:S15632?x-access-code=6fa2ebc53b96e3ae412a8df19187ab41&format=html.

The data sets of Figure 1A and B supporting the results of this article are included as the Additional file 1.

The data sets of Figure 4 supporting the results of this article are included as the Additional file 2.

## Electronic supplementary material

Additional file 1: **A-** **Aligned** ***M. abscessus*** **genome matrix constructed using Mauve software to infer phylogeny using the Neighbor-Net algorithm in the package SplitsTree4. B-** The matrix of binary discrete characters (“0” and “1” for absence and presence, respectively) constructed using the orthologous group data found by orthoMCL to infer phylogeny using the Neighbor-Net algorithm in the package SplitsTree4. (XLS 28 KB)

Additional file 2: **The matrix used for Heatmap clusterisation of** ***Mycobacterium abscessus*** **type VII secretion system compared to** ***Mycobacterium tuberculosis*** **H37Rv.** (XLS 26 KB)
